# Fecal microbiota transplantation in gastrointestinal disorders: time for precision medicine

**DOI:** 10.1186/s13073-020-00757-y

**Published:** 2020-06-30

**Authors:** Nicolas Benech, Harry Sokol

**Affiliations:** 1 Service de Maladies Infectieuses, Hôpital de La Croix-Rousse, Hospices Civils de Lyon, Lyon, France; 2 Université Claude Bernard Lyon 1, Lyon, France; 3 French Group of Fecal Microbiota Transplantation (GFTF; www.gftf.f), Paris, France; 4 Gastroenterology Department, Saint Antoine Hospital, Sorbonne Université, INSERM, Centre de Recherche Saint-Antoine, CRSA, AP-HP, F-75012 Paris, France; 5 INRA, UMR1319 Micalis & AgroParisTech, Jouy-en-Josas, France; 6 Paris Center for Microbiome Medicine (PaCeMM) FHU, Paris, France; 7 AP-HP Fecal Microbiota transplantation Center, Saint Antoine Hospital, Paris, France; 8 Service de Gastro-entérologie, Hôpital Saint-Antoine, 184 Rue du Faubourg Saint-Antoine, 75571 Paris Cedex 12, France

**Keywords:** Fecal microbiota transplantation, Precision medicine, Inflammatory bowel diseases, Irritable bowel syndrome

## Abstract

Fecal microbiota transplantation (FMT) has demonstrated efficacy in treating inflammatory bowel diseases and irritable bowel syndrome in an increasing number of randomized controlled trials. Recently published data gives striking insights into the factors associated with FMT success paving the road for the use of precision medicine in gastrointestinal disorders.

The gut microbiota is now known to be an essential co-factor in the pathophysiology of many diseases. In particular, inflammatory bowel diseases (IBD) and irritable bowel syndrome (IBS) are associated with alteration of the gut microbiota, which contributes to disease onset and maintenance. Therefore, reshaping the gut microbiome using fecal microbiota transplantation (FMT) is an attractive strategy to restore appropriate host-microbiota crosstalk.

Fecal microbiota transplantation consists of the transfer of the fecal microbial ecosystem of a healthy donor to a recipient to induce therapeutic effects. Stool preparations are usually administrated through frozen capsule, enema, colonoscopy, or duodenal infusion. As crude as it might appear, this is, nowadays, the only microbe-based therapy that allows the engraftment of a complex ecosystem into the gut.

Although FMT has been used in clinical practice for many years, many unknowns remain on its precise mode of action and long-term safety. Moreover, the role of the gut microbiota is not the same in different diseases, indicating the need for disease-specific approaches for donor selection and FMT procedure. Recently published data has given insights into the factors associated with FMT efficacy, paving the road for precision medicine in its use in gastrointestinal disorders.

## FMT in gastrointestinal disorders: at the edge of a new era

FMT has proven robust efficacy in the treatment of recurrent CDI (rCDI) and is now recommended with a high level of evidence. CDI is mostly an ecologically driven disease characterized by a loss of gut microbiota barrier properties. In such a situation, FMT would act as a “reset” to restore the gut microbiota richness and diversity to prevent *Clostridioides difficile* growth and pathogenicity. Indeed, one or two FMT is sufficient to cure rCDI in 90% of cases. Efficacy data in other indications are much more recent.

Four randomized controlled trials (RCT) evaluating the ability of FMT to induce steroid-free remission in ulcerative colitis have been published. Three studies reported a higher rate of remission at week 8 or 7 within the FMT group with differences of efficacy compared to placebo ranging from 19 to 23%, whereas one study showed a non-significant trend in favor of FMT. Protocols differed widely among trials regarding the number of FMT per patient (ranging from 2 to 41) and the route of administration (colonoscopy, enemas, naso-duodenal infusion) [1].

In Crohn’s disease, only one RCT evaluating FMT has been published with an original design combining immunological and microbial intervention: remission was first induced by corticosteroids, and one FMT was then performed through colonoscopy. Although it was a small proof-of-concept study, prolonged remission was reported for patients with successful engraftment of donor microbiota [2].

In IBS, a heterogeneous disease characterized by chronic abdominal pain and altered bowel habits, 2 RCT showed significant improvement after FMT [3, 4], while three others were negatives [5–7]. However, characteristics of IBS patients, donors (pooled vs single), and procedures in terms of route of administration and number of infusions (ranging from 1 to 75) were highly variable.

However, despite increasing evidence of efficacy in IBS and IBD, discrepancies between results from one trial to another and heterogeneity within the protocols support the need for further data to establish standards before FMT can be used in routine clinical practice.

## The right donor for the right patient

Recent data suggest that FMT effectiveness for IBS and IBD differs according to the initial recipient microbiota composition, donor microbiota composition, and host immune factors. When considering FMT in complex diseases, one has to keep in mind that gut microbiota is only one factor among others that contribute to pathogenesis. Furthermore, its weight in pathophysiology varies among different diseases, from essential in CDI to maybe more variable in IBS and IBD and likely anecdotal in other conditions. Therefore, the definition of the right donor should depend on the disease. In UC, Moayyedi and colleagues reported a higher remission rate in the recipients of donor B’s stools, which were enriched in members of the Lachnospiraceae family and *Ruminococcus* genus. In another study, higher amounts of *Bacteroides* and *Streptococcus* in donors were associated with positive and negative outcomes, respectively.

Similarly, in IBS, significant improvement was obtained after FMT from a single highly selected donor based on clinical and microbial criteria [4]. Indeed, in this work, the donor was a healthy athletic non-smoking young adult, with a balanced diet who was born by vaginal delivery and whose gut microbiota was enriched in species associated with a healthy condition. Thus, high diversity and the presence of anti-inflammatory bacteria may be important parameters to take into account when selecting donors. However, species of interest may vary between IBS and IBD.

Host factors should also be taken into account when considering eligibility for FMT. Recent data suggest that patients with UC responding to FMT exhibit higher bacterial species richness and *Candida* abundance at baseline, before FMT. Additionally, 15 specific fecal metabolites at baseline were also associated with FMT efficacy and may constitute predictive markers of efficacy [8, 9]. We also reported in CD that FMT failure was associated with baseline enrichment in members of the *Gamma*-*proteobacteria* class such as *Klebsiella*, *Actinobacillus*, and *Haemophilus*, in accordance with previous findings in UC [2, 8]. Altogether, these results suggest that the baseline recipient microbiota may influence the success of donor microbiota engraftment and, consequently, the expected clinical outcome.

Furthermore, as in organ transplantation, donor-recipient microbiota compatibility factors may influence FMT effectiveness. Indeed, it has been shown that donor strains are more likely to colonize the gut if the same species were already present within the recipient microbiota [10].

Patients with IBS and IBD exhibit high heterogeneity in terms of clinical presentation, disease severity, and response to treatment. Moreover, the response to a given treatment can change over time in the same patient. With FMT also, precision medicine strategies should be developed to tailor the therapeutic intervention to each patient. Thus, future FMT clinical trials should include precise characterization of donor and recipient microbiota to identify which factors are associated with disease control and FMT efficacy, paving the way towards precision medicine.

## FMT as maintenance therapy and the need for precise post-FMT monitoring

In IBD and IBS, FMT efficacy is associated with significant donor microbial engraftment, supporting the evaluation of post-FMT recipient microbiota to predict clinical efficacy. In IBS, post-FMT microbiota in responders was characterized by higher amounts of *Eubacterium biforme*, *Lactobacillus* spp., and *Alistipes* spp. and lower amounts of *Bacteroides* spp. [4]. In UC, patients in remission after FMT exhibited an increased abundance of *Eubacterium hallii*, *Roseburia inulinivorans*, *Eggerthella* species, and *Ruminococcus bromii* and a shift in the metabolome towards short-chain fatty acid (SCFA) biosynthesis and secondary bile acids conversion [8]. In contrast, an increase in *Fusobacterium gonidiaformans*, *Sutterella wadsworthensis*, *Escherichia* species, and biosynthesis pathways of heme and lipopolysaccharide were associated with FMT failure. If such predictive markers are confirmed, they might be used to identify patients who will likely need another FMT or a different type of therapeutic strategy earlier.

As host factors involved in disease pathophysiology (genetic variant and environmental factors) will persist after FMT, the effects are likely to be transient with a drift of the recipient microbiota back towards the baseline pathological state. Monitoring specific microbial taxa (*Faecalibacterium*, *Ruminococcus*, *gammaproteobacteria*, *Lactobacillus*, *Candida*), specific metabolites (SCFA, secondary bile acids) in combination with host immune response (anti-*Candida* IgG, fecal calprotectin) could allow clinicians to anticipate disease relapse and define the timing for additional FMT.

Indeed, maintenance therapy using recurrent FMT may be a relevant approach in addition to diet interventions or classical pharmaceutical molecules.

However, new safety issues may be associated with repetitive FMT. Besides the increased risk of infection by undetected or unknown pathogens, the theoretical risk of transmitting a “non-transmissible” microbiota-related disease might become more tangible with iterative gut microbiota transfer for chronic diseases.

## Precision medicine applied to FMT in gastrointestinal disorders

**Fig. 1:**
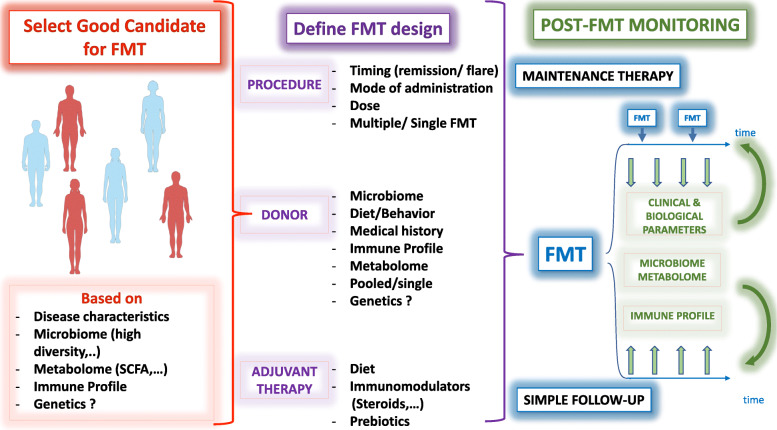
The potential of fecal microbiota transplantation (FMT) precision medicine for gastrointestinal disorders. Left panel: candidates for FMT should be selected on clinical, microbial, immune, and metabolic parameters. Middle panel: FMT design should be tailored to each patient and include the type and timing of the FMT procedure, the criteria for the donor selection, and the potential use of adjuvant therapy. Right panel: patients should be monitored after FMT to determine effectiveness, gauge immune, and microbiome parameters and adjust the therapeutic strategy accordingly. SCFA, short-chain fatty acid

In light of the above results, a personalized approach would be necessary at all steps of the FMT process (Fig. 1). First, candidates to FMT should be selected on predefined clinical, microbial, immune, and metabolic parameters from the blood and stool analysis. The precise definition of such parameters will likely depend on the disease. Second, the FMT design, including the procedure itself, the donor’s characteristics, and the adjuvant therapy, should be established depending on the recipient’s features and the disease to ensure donor’s microbiota engraftment and FMT efficacy. After FMT, specific monitoring should be performed and might include clinical and biological markers of effectiveness, but also immune parameters and microbiome composition and function. All these personalized medicine steps are mandatory to leverage the powerful therapeutic potential of FMT.

## Conclusions

FMT is now at the edge of a new era. Although an increasing amount of data suggests a clinical efficacy in IBS and IBD, precise characterization of patient and donor profiles associated with therapeutic success is now mandatory to define to whom, when, and how physicians should offer FMT.

## Data Availability

Not applicable

## Abbreviations

rCDI: Recurrent *Clostridioides difficile* infection
FMT: Fecal microbiota transplantation
IBD: Inflammatory bowel diseases
IBS: Irritable bowel syndrome
SCFA: Short-chain fatty acid

## References

[1] Imdad A, Nicholson MR, Tanner-Smith EE, Zackular JP, Gomez-Duarte OG, Beaulieu DB (2018). Fecal transplantation for treatment of inflammatory bowel disease. Cochrane Database Syst Rev.

[2] Sokol H, Landman C, Seksik P, Berard L, Montil M, Nion-Larmurier I (2020). Fecal microbiota transplantation to maintain remission in Crohn’s disease: a pilot randomized controlled study. Microbiome.

[3] Johnsen PH, Hilpüsch F, Cavanagh JP, Leikanger IS, Kolstad C, Valle PC (2018). Faecal microbiota transplantation versus placebo for moderate-to-severe irritable bowel syndrome: a double-blind, randomised, placebo-controlled, parallel-group, single-centre trial. Lancet Gastroenterol Hepatol.

[4] El-Salhy M, Hatlebakk JG, Gilja OH, Bråthen Kristoffersen A, Hausken T (2020). Efficacy of faecal microbiota transplantation for patients with irritable bowel syndrome in a randomised, double-blind, placebo-controlled study. Gut..

[5] Aroniadis OC, Brandt LJ, Oneto C, Feuerstadt P, Sherman A, Wolkoff AW (2019). Faecal microbiota transplantation for diarrhoea-predominant irritable bowel syndrome: a double-blind, randomised, placebo-controlled trial. Lancet Gastroenterol Hepatol..

[6] Holster S, Lindqvist CM, Repsilber D, Salonen A, de Vos WM, König J (2019). The effect of allogenic versus autologous fecal microbiota transfer on symptoms, visceral perception and fecal and mucosal microbiota in irritable bowel syndrome: a randomized controlled study. Clin Transl Gastroenterol.

[7] Halkjær SI, Christensen AH, Lo BZS, Browne PD, Günther S, Hansen LH (2018). Faecal microbiota transplantation alters gut microbiota in patients with irritable bowel syndrome: results from a randomised, double-blind placebo-controlled study. Gut..

[8] Paramsothy S, Nielsen S, Kamm MA, Deshpande NP, Faith JJ, Clemente JC (2019). Specific bacteria and metabolites associated with response to fecal microbiota transplantation in patients with ulcerative colitis. Gastroenterology.

[9] Leonardi I, Paramsothy S, Doron I, Semon A, Kaakoush NO, Clemente JC, et al. Fungal trans-kingdom dynamics linked to responsiveness to fecal microbiota transplantation (FMT) therapy in ulcerative colitis. Cell Host Microbe. 2020;27(5):823–829. 10.1016/j.chom.2020.03.006 PMC8647676 32298656

[10] Li SS, Zhu A, Benes V, Costea PI, Hercog R, Hildebrand F (2016). Durable coexistence of donor and recipient strains after fecal microbiota transplantation. Science..

